# Low ALT levels and a new diagnosis of congestive heart failure following COVID-19 infection

**DOI:** 10.3389/fcvm.2025.1470239

**Published:** 2025-08-05

**Authors:** Asher Shafrir, Gil Dagan, Ronny Alcalai, David Leibowitz

**Affiliations:** ^1^ Jerusalem District, Meuhedet Health Maintenance Organization, Tel Aviv, Israel; ^2^ Department of Gastroenterology and Hepatology, Hadassah-Hebrew University Medical Center, Jerusalem, Israel; ^3^ Faculty of Medicine, Hebrew University of Jerusalem, Jerusalem, Israel; ^4^ Heart Institute, Hadassah Medical Center and Faculty of Medicine, Hebrew University of Jerusalem, Jerusalem, Israel

**Keywords:** COVID-19, congestive heart failure, ALT, frailty, sarcopena

## Abstract

**Aims:**

COVID-19 infection may result in complications including congestive heart failure (CHF). It is vital to identify factors associated with CHF post COVID so as to improve outcomes. The aim of this study was to determine whether baseline low alanine transaminase (ALT) levels are associated with new diagnosis of CHF following infection with COVID-19.

**Methods and results:**

The study was a retrospective cohort study of patients in the Meuchedet Health Fund database. The analysis was performed on all subjects aged 18 years and older who tested positive for SARS-CoV-2 and had ALT levels measured prior to infection. Patients were excluded if diagnosed with congestive heart failure or cirrhosis prior to COVID-19, or if they died within 30 days of SARS-CoV-2 contraction. The study endpoint was a new diagnosis of CHF as recorded in the subject's electronic medical record.

**Results:**

131,953 adult patients infected with COVID were included in the cohort. Of them, 205 patients (0.16%) were diagnosed with CHF following COVID-19 infection. The occurrence of CHF was significantly higher in the low ALT group (0.34% vs. 0.14%, *p*- value < 0.001). This difference was more prominent when analyzing patients aged 50 and older (1.4% vs. 0.35, *p*-value < 0.001). In a multivariate logistic regression, pre-morbid low ALT remained significantly associated with the occurrence of post COVID-19 CHF (OR—1.95, 95% CI −1.03 to 2.53).

**Conclusions:**

This study demonstrates that low ALT levels prior to infection with COVID-19 is associated with a new diagnosis of CHF following infection. Patients with low ALT levels prior to COVID-19 infection should have cardiovascular complaints post COVID carefully assessed.

## Graphical Abstract

**Figure:**
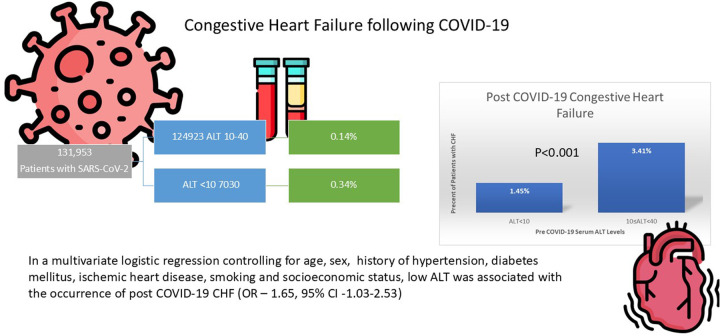

## Introduction

The ongoing coronavirus disease 2019 (COVID-19) pandemic continues to present significant public health challenges. COVID-19 infection may result in significant complications involving the cardiovascular system including myocarditis and new onset congestive heart failure (CHF) through a variety of mechanisms including direct viral injury, inflammatory activation and epicardial or microvascular occlusive disease (1–3). Imaging studies have also demonstrated a high incidence of cardiac abnormalities during and following COVID-19 which may lead to a subsequent diagnosis of CHF (4, 5). Cardiac involvement in COVID-19 may be acute or post acute as part of “long” COVID syndrome (6, 7). Premorbid risk factors for the development of CHF following a diagnosis of COVID-19 remain unclear, however it appears factors such as prolonged inflammatory state, frailty and sarcopenia may play an important role (8). It is vital to identify factors contributing to or predictive of CHF in post COVID patients so as to design preventive strategies to improve outcomes.

Sarcopenia and frailty are increasingly recognized as important contributors to morbidity and mortality in the general population (9). Although the clinical implication of frailty is undoubtably important, many patients are underdiagnosed and the need for effective screening tools are warranted (10).Alanine transaminase (ALT) is an enzyme generally utilized to assess liver damage, however ALT levels are a marker for skeletal muscle mass and low levels are associated with sarcopenia and frailty (11, 12). Previous studies have shown that low ALT levels are associated with worse outcomes in a variety of cardiovascular diseases, including CHF (13, 14). No previous study has examined whether low ALT levels prior to COVID-19 disease are predictive of cardiovascular complications following infection, a question with important clinical implications regarding follow up and treatment of these patients. The hypothesis of this study was that baseline low ALT levels are associated with a new diagnosis of CHF following infection with COVID-19.

## Methods

Meuhedet health maintenance organization (HMO) is one of Israel's state-mandatory HMOs. It is the third largest HMO in Israel and serves over than 1,300,000 individuals. Meuhedet's computerized database includes real-time input from all physician visits, medical diagnoses, laboratory results, hospitalizations, and dispensing data on prescription and over-the-counter medications. The database has been validated, and multiple studies, including those pertaining to CHF, have demonstrated its utility (15, 16). Data from 1,072,521 patients from the HMO was collected as part of a prospective study following all HMO patients who underwent SARS-CoV-2 testing from March 2020 to 31 April 2022. We gathered data from the electronic medical record including diagnoses documented in the medical record at any point prior to SARS-CoV-2 testing and blood tests in the prior year. Data regarding hospitalization in a medical ward or ICU following SARS-CoV-2 testing was collected. In addition, medical diagnoses added to the EMR after COVID-19 were collected, with blood tests taken at least 30 days after the first SARS-CoV-2 positive test.

The analysis was performed on all subjects aged 18 years and older who tested positive for SARS-CoV-2. Patients were excluded if they were diagnosed with congestive heart failure or cirrhosis (ICD -9 codes 428, 428.0, 428.1, 428.9, 429) prior to COVID-19, if they died within 30 days of SARS-CoV-2 contraction, or if they were diagnosed with COVID-19 less than two months before the data was collected.

Socioeconomic status was gathered using Points Location Intelligence (https://points.co.il). The index uses Israel Central Bureau of Statistics data according to residency address. The classification uses data regarding average family size, income, educational level, unemployment rate, number of cars per family, and a median age of the population in the geographical unit. The score is on a scale of 1–10, with 1 as the lowest (17).

Similar to other studies, ALT levels were divided into three groups, low (ALT ≤10 IU/L), normal (11–40 IU/L), and high (ALT > 40 IU/L). Similarly to other studies, patients with ALT > 40 were excluded as high ALT levels may be related to liver disease and not to muscle mass (18, 19).

A diagnosis of CHF was defined as a diagnosis recorded in the electronic medical record for the first time following COVID (ICD 9 codes 428–429). This research was conducted in accordance with the Declaration of Helsinki and approved by the research ethics committee and internal review board of Meuhedet HMO (02-24-08-20) on September 2, 2020. The need for informed consent was waived as the data was anonymized, and no intervention occurred.

### Statistical analysis

The characteristics of patients with low ALT were compared to those with normal ALT. Categorical variables were summarized as counts and percentages and were compared using the Chi-square test. Continuous variables were summarized by means and standard deviation (SD) and were compared using the student *t*-test method. The incidence of CHF following COVID-19 was calculated in each ALT group and was compared using the Chi-square test. Multivariate logistic regression was constructed to control for confounders that could mediate the association of low ALT with CHF diagnosis, and odds ratio (OR) and 95% confidence intervals (CI) were reported. In addition, a 3:1 propensity score matched cohort was constructed. Patients were matched according to known risk factors for CHF diagnosis. A *p*-value of less than 0.05 was considered statistically significant in all analyses. Statistical analysis was performed using R software (32).

## Results

**Figure 1:**
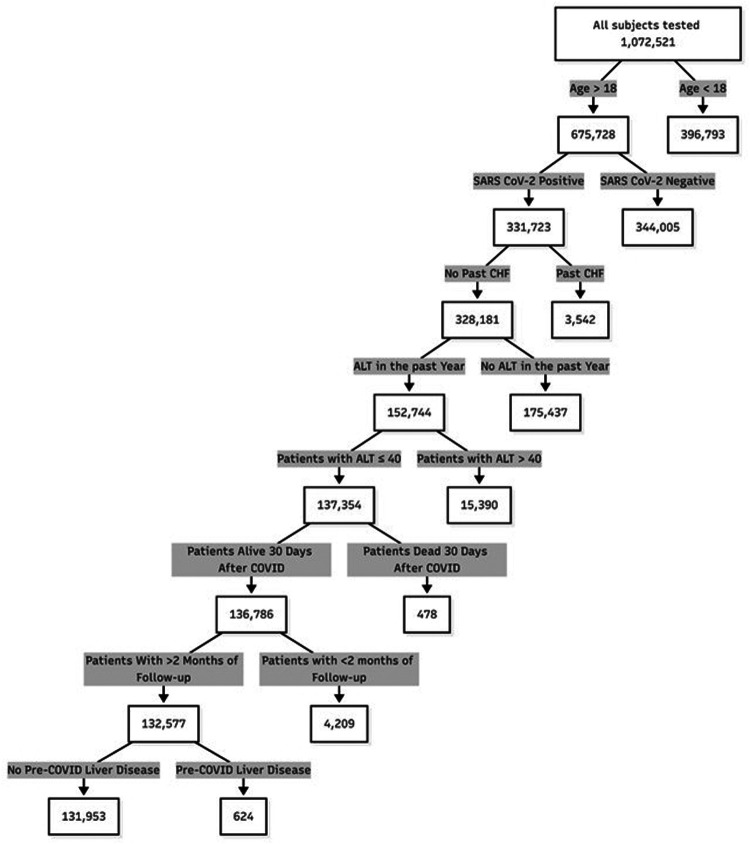
Patient flow chart.

During the study period, 331,723 adult patients were diagnosed with COVID-19. Of these, 152,744 had normal ALT documented within 1 year prior to disease. After excluding patients who died within 30 days of SARS-CoV-2 infection, those with a follow up of less than 2 months, and those with a history of liver disease or CHF, 131,953 were included in the analysis (Figure 1).

Table 1 depicts demographic and clinical characteristics of the study population. Patient with low ALT were significantly younger (40.8 years ± 19.3 vs., 44.9 ± 17.0, *p* < 0.001) and more likely to be female (11.3% vs. 36.3%, *p* < 0.001). Patients with normal ALT had higher BMI (27.0 ± 5.5 vs. 25.6 ± 5.6, *p*-value < 0.001), had higher rates of hypertension (7.7% vs. 6.5%, *p*-value < 0.001) diabetes mellitus (10.1% vs. 8.6% *p*-value < 0.001) and history of ischemic heart disease (3.6% vs. 3.1% *p*-value = 0.011). While the mean BMI was slightly elevated in the low ALT group, a low BMI (<19 kg/m^2^) was significantly higher in this group (7.3% vs. 4.1%, *p*-value < 0.001).

**Table 1:** Clinical characteristics of the patient population.

Variables	Normal ALT	Low ALT	*p*-value
N	124,923	7030	
Age (mean (SD))	44.93 (17.02)	40.80 (19.28)	<0.001
Sex = male (%)	45,388 (36.3)	795 (11.3)	<0.001
BMI (mean (SD))	26.98 (5.54)	25.63 (5.63)	<0.001
Smoking (%)	12,658 (10.1)	579 (8.2)	<0.001
SES (mean (SD))	5.22 (2.03)	4.98 (2.00)	<0.001
Hypertension (%)	9,624 (7.7)	460 (6.5)	<0.001
Diabetes mellitus (%)	12,555 (10.1)	607 (8.6)	<0.001
Ischemic heart disease (%)	4,508 (3.6)	215 (3.1)	0.017

BMI, body mass index; SES, socioeconomic status.

Low ALT was significantly associated with an increased incidence of other markers of frailty and sarcopenia such as low albumin (14% vs. 1.6%, *p* < 0.001) and low creatinine (0.2% vs. 0.005%, *p* ≤ 0.001).

**Figure 2:**
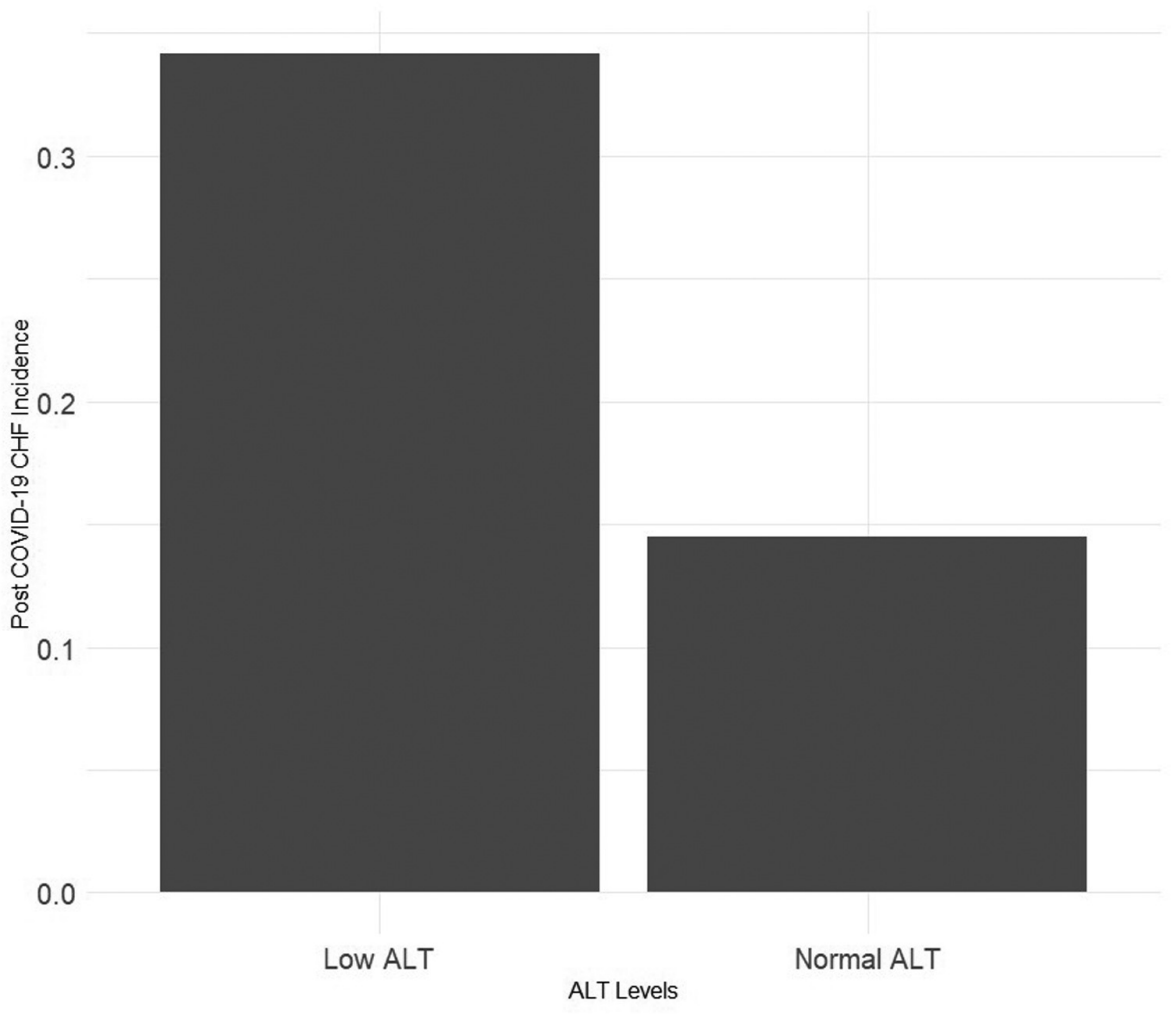
Precent of patients with post COVID-19 congestive heart failure by pre-infection alanine transaminase (ALT) levels.

Of the population analyzed, 205 (0.16%) were diagnosed with CHF following COVID-19 infection. The occurrence of CHF was higher in the low ALT group (0.34% vs. 0.14%, *p*- value < 0.001), Figure 2. This difference was more prominent when analyzing patients aged 50 and older (1.4% vs. 0.35, *p*-value < 0.001).

In a multivariate logistic regression controlling for age, sex, history of hypertension, diabetes mellitus, ischemic heart disease, smoking and socioeconomic status, pre-morbid low ALT remained significantly associated with the occurrence of post COVID-19 CHF (OR—1.64, 95% CI −1.03 to 2.53, see Table 2, graphical abstract).

**Table 2:** Multivariate logistic regression analyzing factors associated with post COVID-19 congestive heart failure.

Variables	Odds ratio	*p*-value	5% CI	95% CI
Age	1.077	<0.001	1.066	1.089
Sex = male	1.309	0.066	0.982	1.747
Hypertension	1.826	<0.001	1.345	2.473
Diabetes mellitus	1.709	<0.001	1.271	2.290
Smoking	1.553	0.050	0.977	2.364
Low ALT	1.646	0.029	1.027	2.529
SES	0.710	<0.001	0.580	0.868
Ischemic heart disease	2.576	<0.001	1.858	3.542

SES, socioeconomic status.

A 1:3 propensity score matched cohort where patients who developed post COVID CHF were matched to patients who did not according to age, sex, history of smoking, hypertension, diabetes and ischemic heart disease was constructed. In this cohort, low ALT was significantly more prevalent in the CHF group compared to subjects who did not develop CHF (11.7% vs. 6.2%, *p*-value = 0.003).

To rule out severe COVID-19 as a mediator of low ALT and post COVID-19 CHF, we performed an additional analysis excluding patients who were hospitalized in ICU at any point (*n* = 288) or in a ward for more than 10 days due to COVID-19 (*n* = 973). In this sub-group, low ALT remained associated with post COVID-19 CHF (0.31% vs. 0.12%, *p*-value < 0.001). A multivariate logistic regression constructed using this cohort showed similar results (OR—2.25, 95% CI 1.36–3.56, *p*-value < 0.001).

### Sensitivity analysis

A sensitivity analysis was performed to examine different cutoffs for low ALT. When using 13 U/L as the cutoff, patients with low ALT had an odds ratio of 1.45, *p* = 0.03 of being diagnosed with post COVID CHF. To exclude survival bias, a sensitivity analysis included patients who died within 30 days of COVID infection. In this cohort, ALT was significantly associated with post COVID CHF (3.3% vs. 1.4% *p* ≤ 0.001). In addition, when excluding patients who died within 90 days of COVID infection, low ALT remained associated with post COVID CHF (2.1% vs. 1.4%, *p* = 0.27).

## Discussion

This innovative study demonstrates for the first time that low ALT levels prior to infection with COVID-19 is associated with a new diagnosis of CHF following infection. While the overall occurrence of CHF was low in our cohort, the incidence in the population over the age of 50 was not negligible and these findings have potentially important clinical implications for management of these patients

Myocardial injury in the setting of COVID-19 infection has been extensively reported since the outbreak of the pandemic and can range from asymptomatic elevation of cardiac biomarkers to fulminant myocarditis and death (20). Potential causes include myocarditis, exacerbation of underlying coronary artery disease, toxic/inflammatory effects, and stress cardiomyopathy (1–3, 21). Risk factors for acute myocardial injury remain unclear but include advanced age, male sex, underlying vascular disease and vascular risk factors and obesity (22). In addition to acute cardiac complications of COVID-19 infection, there are post-acute cardiac sequelae as well (6, 7). While a relatively high incidence of possibly cardiac related symptoms such as dyspnea, chest pain and palpitations have been reported post COVID-19 infection, relatively few of these patients were found to have a cardiovascular diagnosis (23). Our finding of a very low incidence of new diagnosis of CHF post COVID is consistent with these findings. Given the enormous numbers of COVID infections in the general population however, even a small incidence of CHF post infection has important public health ramifications. Risk factors for these complications are not well studied and our finding of an association between a simple measurement of low ALT and a new CHF diagnosis is novel and relevant. Providing an easily measured and inexpensive population-based marker for cardiac complications following COVID infection is an important advance in the care of these patients. It suggests that patients with low ALT levels with COVID should receive more aggressive clinical follow-up with a low threshold for cardiac imaging such as echocardiography to assess cardiac function. In addition, biomarkers of cardiac damage such as troponin and brain natriuretic peptide may be helpful in this population and this is an important area for future study. Early diagnosis of cardiac dysfunction offers the opportunity for early medical therapy such as ACE inhibitors to prevent clinical CHF, an intervention shown to be clinically effective in other populations such as post MI patients. Frailty is a potentially reversible process and interventions such as cardiac rehabilitation to reduce frailty have shown benefit in patients with known CHF (24). Therefore, treatment to reduce frailty in susceptible COVID patients may reduce the development of CHF.

**Figure 3:**
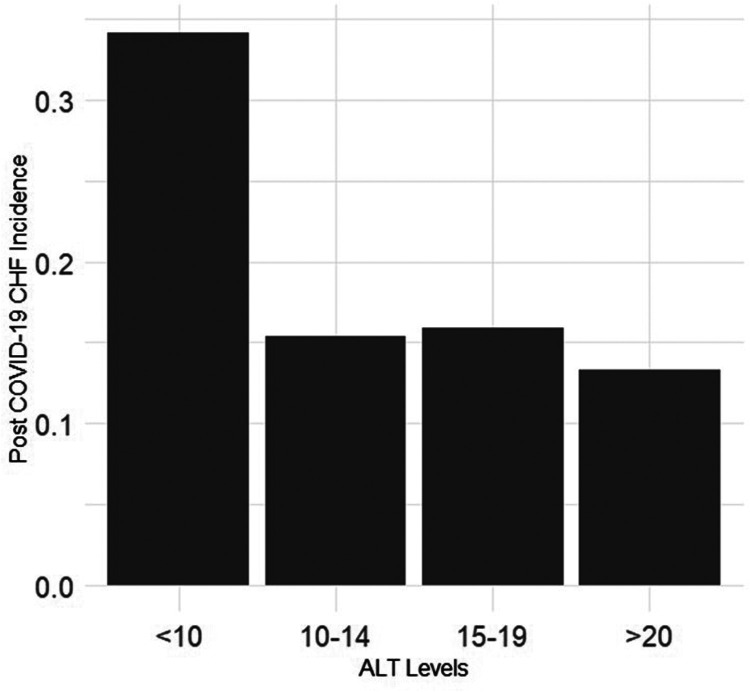
Precent of patients post-COVID congestive heart failure according to pre-COVID alanine transaminase levels.

Sarcopenia and frailty are increasingly recognized as important contributors to general as well as cardiovascular mortality in the population (9, 25). Frailty has been associated with mortality in elderly patients with COVID-19 as well (26). Alanine transaminase (ALT) is an enzyme generally utilized to assess liver damage, however ALT levels are a marker for skeletal muscle mass and low levels are associated with sarcopenia and frailty (11, 12). Previous studies have shown that low ALT levels are with worse outcomes in a variety of cardiovascular diseases including in a broad range of patients with chronic CHF (13, 14, 27). Our study expands these findings to a post-COVID-19 population. According to different studies, low ALT values between 10 and 17 are considered low (13, 14, 18). While the exact cutoff can be disputed, there is clearly an increase in post-COVID CHF incidence as ALT levels decrease (Figure 3). The mechanisms of the association between low ALT levels and susceptibility to CHF post COVID remains speculative. Frailty and sarcopenia have been associated with circulating inflammatory cytokines and a pro-inflammatory state which in combination with the multiple physiologic insults of COVID-19 infection may lead to an increased incidence of clinical CHF (28). Decreased protein intake and prolonged inactivity which may be seen in COVID illness particularly in the elderly have been associated with decreased antioxidant activity, a finding present in patients with frailty, sarcopenia and CHF as well (29).Another mechanism common to both frailty and CHF include cellular senescence which has been reported as a complication of COVID infection as well (30).

The major limitation of the study is its retrospective nature and the inherent inability to adjust for all possible confounders. In particular other factors which may influence ALT levels such as genetic factors and environmental exposures could not be accounted for given the limitations of the database. We used length of hospitalization and ICU stay as surrogate markers for COVID severity. Data regarding blood tests and need of oxygen in the hospital is not accessible in the database. Details of possible cardiac complications during acute COVID infection were not available in the database. We excluded patients who died within 30 days of infection, presumably limiting the inclusion of patients with fulminant cardiac disease secondary to COVID-19. In addition, the post COVID diagnosis of CHF was assessed from medical records, similar to other studies from the HMO (15, 16). While in different centers, the administrative code of CHF was effective in identifying patients who truly suffer from CHF (31), the validity of the diagnosis cannot be assessed with certainty in our cohort. According to IRB regulations, we do not have access to individualized patient records, natriuretic peptide is not tested regularly in the HMO, and echocardiography data could not be readily accessed in the database. We were also unable to determine that there was no evidence of pre-COVID subclinical CHF becoming overt following COVID. Furthermore, the use of medication that can affect cardiac function prior to COVID was incomplete; therefore, we could not assess interactions between different drugs and post-COVID CHF.

In conclusion, our study demonstrates for the first time that low ALT levels prior to infection with COVID-19 were independently associated with a new diagnosis of CHF following infection, particularly in patients over 50 years of age. This simple, easily obtainable laboratory test may be helpful in assessing the risk of CHF post COVID-19 and facilitating the clinical evaluation of patients with cardiovascular complaints following COVID infection.

## Data Availability

The datasets presented in this article are not readily available because there are ethical restrictions on sharing our dataset as the data contains potentially identifying patient information. These restrictions were imposed by the Ethics Committee of Meuhedet HMO which owns the data. Requests to access the datasets should be directed to liron.y3@meuhedet.co.il.
